# Amphiregulin activates regulatory T lymphocytes and suppresses CD8^+^ T cell-mediated anti-tumor response in hepatocellular carcinoma cells

**DOI:** 10.18632/oncotarget.5171

**Published:** 2015-10-06

**Authors:** Chun-Hui Yuan, Xiao-Ming Sun, Cheng-Liang Zhu, Shao-Ping Liu, Long Wu, Hao Chen, Mao-Hui Feng, Ke Wu, Fu-Bing Wang

**Affiliations:** ^1^ Department of Laboratory Medicine, Zhongnan Hospital of Wuhan University, Wuchang District, Wuhan 430071, P.R. China; ^2^ Department of Immunology, School of Basic Medical Sciences, Wuhan University, Wuchang District, Wuhan 430071, P.R. China; ^3^ The State Key Laboratory of Virology, College of Life Sciences, Wuhan University, Wuchang District, Wuhan 430072, P.R. China; ^4^ Hubei Key Laboratory of Tumor Biological Behaviors & Hubei Cancer Clinical Study Center, Zhongnan Hospital of Wuhan University, Wuchang District, Wuhan 430071, P.R. China; ^5^ Department of Oncology, Renmin Hospital of Wuhan University, Wuchang District, Wuhan 430060, P.R. China; ^6^ Department of Oncology, Zhongnan Hospital of Wuhan University, Wuchang District, Wuhan 430071, P.R. China; ^7^ Animal Experiment Center of Wuhan University/Animal Biosafety Level-III laboratory, Wuchang District, Wuhan 430071, P.R. China

**Keywords:** hepatocellular carcinoma, amphiregulin, CD4^+^ regulatory T cells, CD8^+^ T cells

## Abstract

CD8^+^ T cell-mediated immune response plays an important role in inhibiting progression of hepatocellular carcinoma (HCC). For strategic immunotherapy, it is critical to understand why some of the tumor cells escape from this immune attack. In this study, we investigated how HCC cells alter endogenous anti-tumor immunity and their related signaling pathways. We found that HCC cells, both *in vitro* and *in vivo*, substantially secret and express amphiregulin (AR). AR in turn activates immunosuppressive function of intratumoral CD4^+^Foxp3^+^ regulatory T cells (Tregs), a major inhibitor of CD8^+^ T cells. Using either lentiviral siRNA, or AR neutralizing antibody, we blocked the expression and function of AR to test the specificity of AR mediated activation of Tregs, Biochemical and cell biology studies were followed and confirmed that blocking of AR inhibited Tregs activation. In addition, we found that AR can trigger the activation of rapamycin complex 1(mTORC1) signaling in Tregs. The mTORC1 inhibitor rapamycin treatment led to compromise Treg function and resulted in enhancing anti-tumor function of CD8^+^ T cells. Blocking AR/EGFR signaling in Tregs with Gefitinib also enhanced anti-tumor immunity and decreased tumor size in a mouse xenograft tumor model. Taken together, our study suggested a novel mechanism of functional interaction between HCC and Tregs for regulating anti-tumor function of CD8^+^ T cells.

## INTRODUCTION

Hepatocellular carcinoma (HCC) is the 3rd most common cause of cancer-related death worldwide. Spontaneous immune responses including T-cell responses [1] and humoral responses to different tumor-associated antigens [2] have been suggested to inhibit the tumor growth of HCC. It is recognized that IFN-γ-producing CD8^+^ T cells play an important role in inhibiting and killing tumor cells and impeding tumor growth. However, not all T cells are anti-tumor effector immune cells. A subpopulation of CD4^+^ T cells that express CD25 and the master transcriptional factor Foxp3, termed regulatory T cells (Tregs), play a crucial role in promoting tumor growth and progress by inhibiting anti-tumor CD8^+^ T cells [3–5]. Although previous observations have demonstrated the function of Tregs in inhibiting anti-tumor CD8^+^ T cells in HCC [6–9], the underlying cellular and molecular mechanisms concerning the activation of Tregs still remain largely unknown. Most of the previous studies focused on either how Tregs suppress tumor-associated antigen (TAA) specific effector T cell function, or how Tregs regulate tumor-associated inflammation. The cellular and molecular mechanisms underlying the modulation of Tregs activity has been overlooked.

Recently, amphiregulin (AR) has been shown to activate Tregs by binding to the epidermal growth factor receptor (EGFR) on Tregs surface [10, 11]. Interestingly, AR has been found to be present in a number of tumor tissues including HCC [12–17], suggesting a potential crosstalk between cancer cells and Tregs through AR for regulating tumor immunity. In this study, we investigated the role of AR produced by HCC cells and its function on regulating Tregs. We found that AR was over expressed in both cultured HCC cells and HCC mouse xenografts. In addition, we found that intratumoral Tregs expressed higher EGFR than their splenic counterparts. The expression of CTLA-4 (cytotoxic T-lymphocyte-associated protein 4) and ICOS (Inducible T-cell COStimulator) in intratumoral Tregs was up-regulated, and the Tregs-mediated suppression of CD8^+^ T cell function was enhanced in HCC. HCC-derived AR promoted Tregs function partially through activation of the rapamycin complex 1 (mTORC1) signaling pathway. Knockdown of AR expression in HCC cells or blockage of EGFR signaling in Tregs enhanced CD8^+^ T cell-mediated anti-tumor response. These data suggested a novel mechanism by which anti-tumor immunity is modulated in HCC.

## RESULTS

### HCC cells overexpress AR

**Figure 1:**
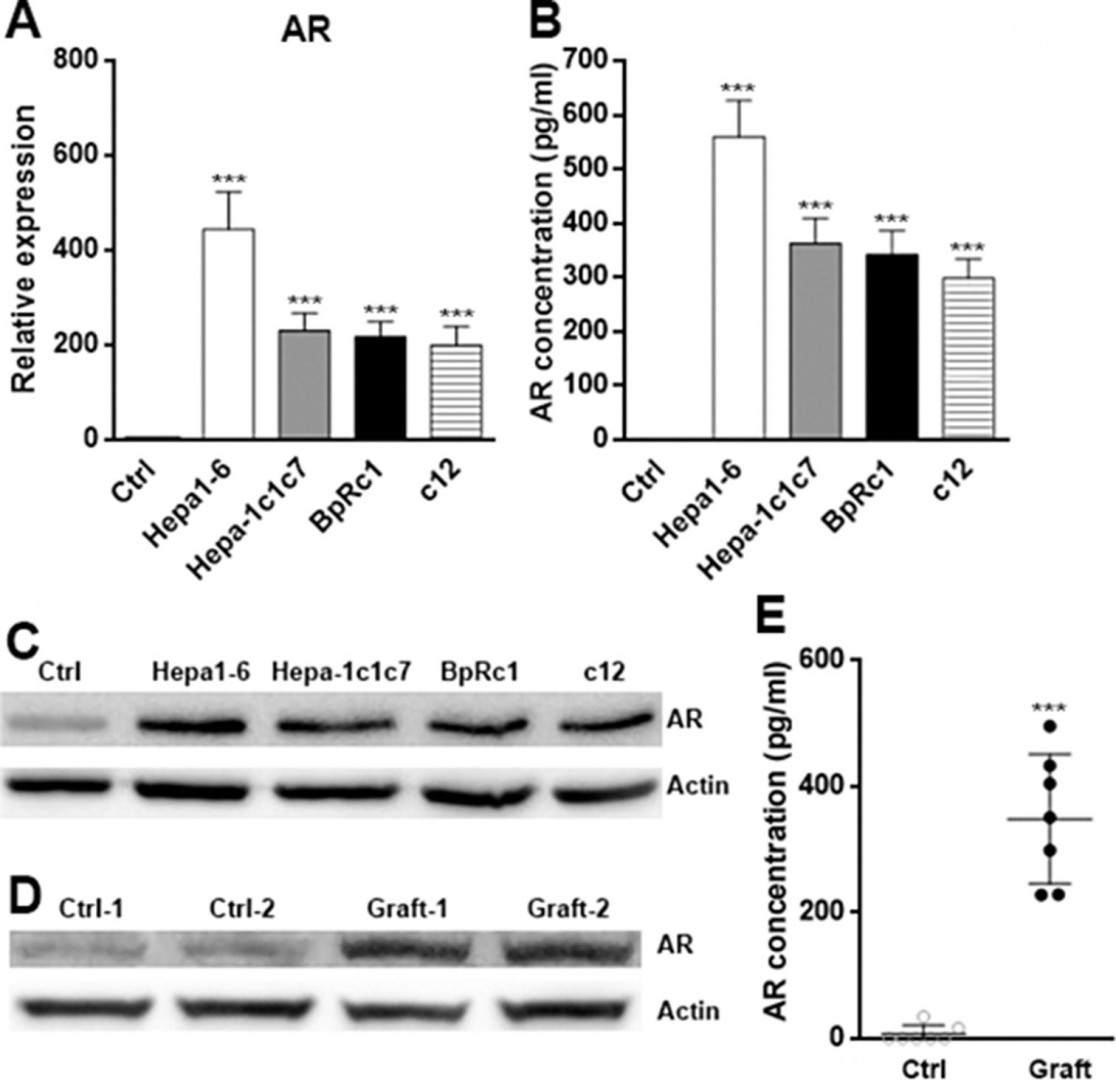
Expression of AR in HCC cell lines **A-C.** Normal murine hepatocytes, murine HCC cell lines Hepa1–6, Hepa-1c1c7, BpRc1 and c12b were cultured *in vitro*. AR expression was determined by qRT-PCR (A), ELISA (B) and Western blotting (C) Ctrl, normal hepatocytes. **D-E.** AR expression in normal liver tissue and Hepa1–6 xenografts in Rag1^−/−^ mice were determined by Western blotting (D) and ELISA (E) Ctrl, normal liver tissue; Graft, Hepa1–6 xenograft. *N* = 8 per group. Data presented as mean ± SD. ***P < 0.001 compared with ctrl.

Previous studies have demonstrated that AR is highly expressed in HCC and other tumor cells [16, 17, 21–23]. To confirm these observations, we assessed expression of AR in multiple HCC cell lines. Consistent with previous reports, all HCC cell lines including Hepa1–6, Hepa-1c1c7, BpRc1 and c12 has higher AR expression as compared to that in normal hepatocytes, as demonstrated by Western blotting and real-time PCR analysis (Figure 1A∼1C). Among these cell lines, Hepa1–6 has the highest AR expression. Based on this result, we used Hepa1–6 to test the AR function in regulating tumor immunity in the following experiments. To evaluate AR expression *in vivo*, Hepa1–6 cells were injected s.c. into Rag1^−/−^ mice to establish a xenograft tumor model. We found that AR expression in the xenografts remains high as determined by Western blotting and ELISA (Figure 1D, 1E). Our data indicated that HCC cells overexpress AR both *in vitro* and *in vivo*.

### Phenotype of intratumoral Tregs

**Figure 2:**
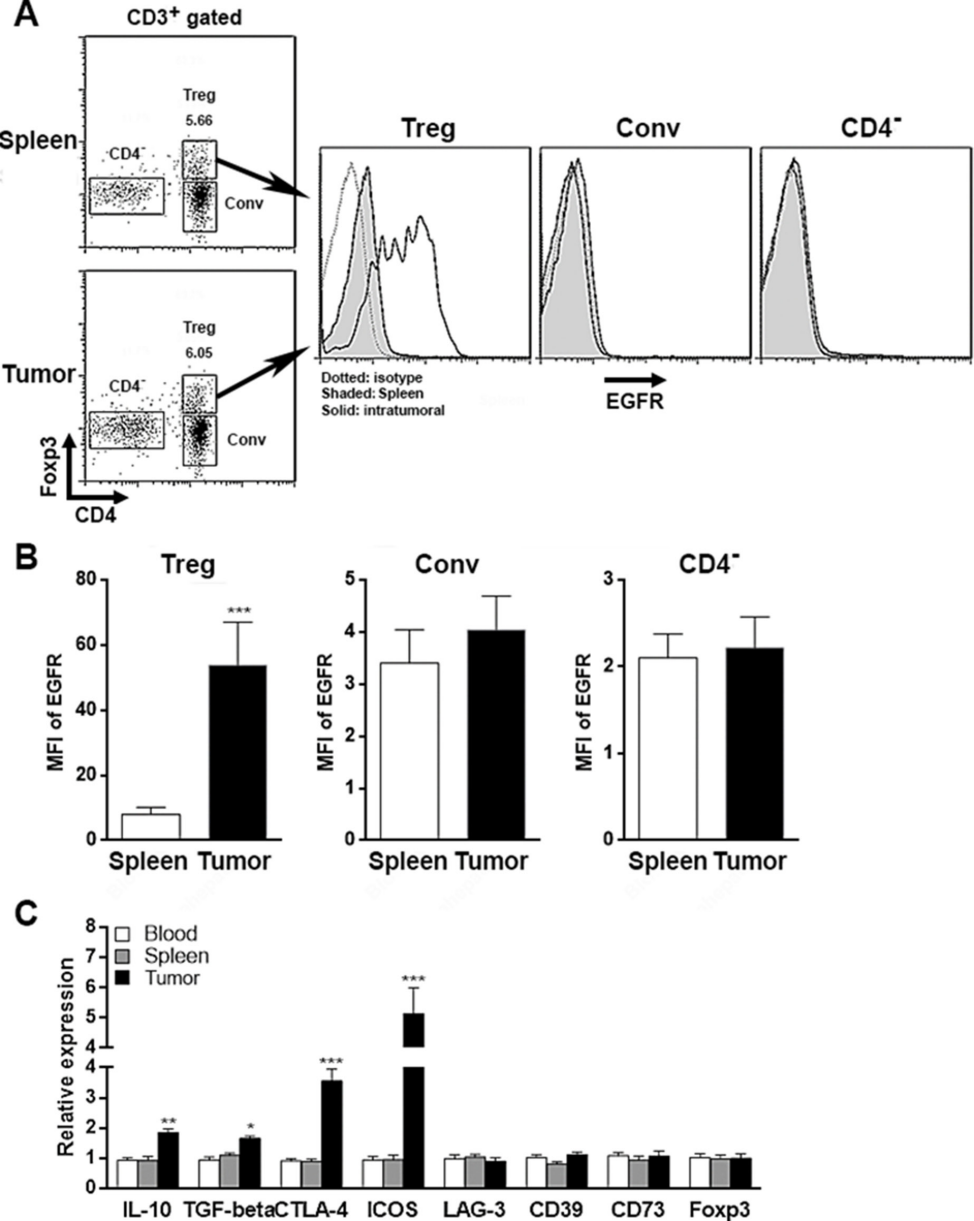
Phenotype of intratumoral Tregs **A.** Detection of EGFR on T cells isolated from Rag1^−/−^ mouse spleens and Hepa1–6 xenografts after adoptive transfer of C57BL/6J splenic T cells. Each T subset was gated for analysis of EGFR expression. Left panel, gating strategies for T subsets. Numbers in the plots were the percentages of Tregs in total T cells. Right panel, representative histograms of EGFR staining. Spleen, splenic T cells; tumor, intratumoral T cells. Conv, CD4^+^ conventional T cells. CD4^−^, CD4^−^ T cells (mostly CD8^+^ T cells). **B.** Statistical analysis on the mean fluorescent intensity (MFI) of EGFR staining. **C.** Signature gene expression in Tregs isolated from blood, spleens and tumor xenografts was determined by qRT-PCR. *N* = 8 per group. Data presented as mean ± SD. *p < 0.05; **p < 0.01; ***p < 0.001 compared with splenic Tregs.

To analyze the adaptive anti-HCC immunity, 2 × 10^7^ splenic CD3^+^ T cells were isolated from C57BL/6J mice and were adoptively transferred into HCC-inoculated mice twice a week from day 7 to day 28 after inoculation. Then T cells were isolated from the xenografts and spleens and were analyzed for their phenotype. Splenic T cells and intratumoral T cells contained similar proportions of CD4^+^Foxp3^+^ Tregs, CD4^+^CD25^−^ conventional T cells and CD4^−^ T cells. However, as demonstrated by flow cytometry analysis probing with EGFR antibody, the EGFR expression was up-regulated only in intratumoral Tregs, not in splenic Tregs (Figure 2A, 2B). In addition, mRNA levels of IL-10, TGF-β, CTLA-4 and ICOS were increased in intratumoral Tregs (Figure 2C), indicating that intratumoral Tregs exhibited an activated phenotype. This result implied that HCC might contribute to the activation of Tregs.

### HCC cells alter the Treg phenotype through AR

**Figure 3:**
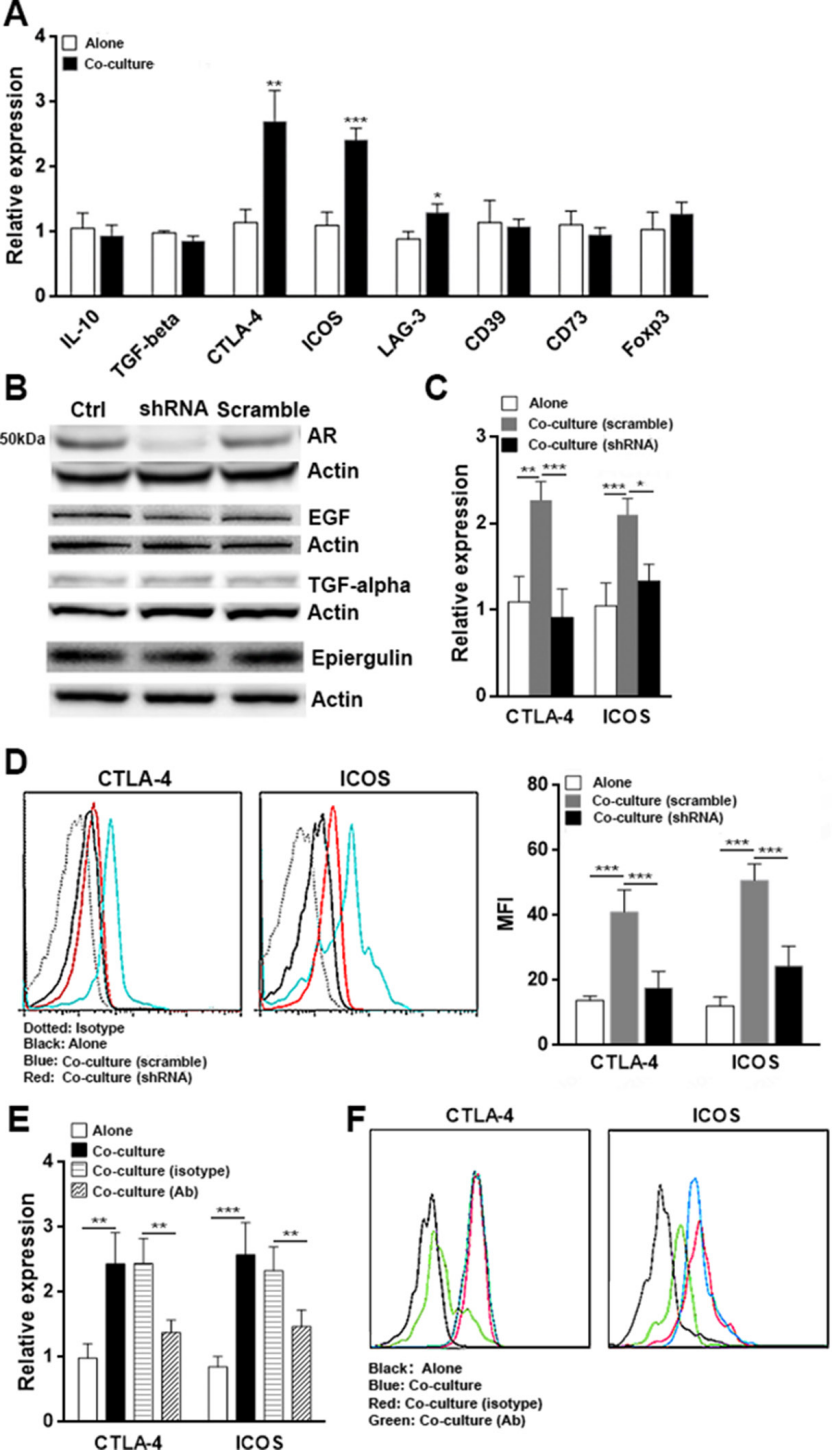
HCC cells alter Treg phenotype through AR **A.** Intratumoral Tregs were enriched from intratumoral mononuclear cells as described in Materials and methods. Tregs were co-cultured with Hepa1–6 cells in Transwell plates for 24 h, followed by determining Tregs signature gene expression using qRT-PCR. Alone, Tregs cultured alone; Co-culture, Tregs cultured with Hepa1–6 cells. **B.** Tranfection of Hepa1–6 cells with AR shRNA-containing lentivirus (LV-ARsh) down-regulated AR protein level. Ctrl, non-transfected cells; shRNA, cells transfected with AR shRNA-containing lentivirus; Scramble, cells transfected with scramble shRNA-containing lentivirus (LV-scramble). This is a representative of two independent experiments. (C–D) Intratumoral Tregs were co-cultured with Hepa1–6 cells transfected with LV-ARsh or LV-scramble. Expression of CTLA-4 and ICOS in Tregs was analyzed by qRT-PCR **C.** and flow cytometry **D.** Left panel of (D), representative histograms. Right panel of (D), Statistical analysis for the mean fluorescent intensity (MFI) of CTLA-4 and ICOS. **E-F.** Tumor-infiltrating Tregs were co-cultured with Hepa1–6 cells in the presence of AR neutralizing antibody or polyclonal goat IgG. Expression of CTLA-4 and ICOS in Tregs was analyzed by qRT-PCR (E) and flow cytometry (F) Alone, Tregs cultured alone; Co-culture (scramble), Tregs cultured with LV-scramble-transfected Hepa1–6; Co-culture (shRNA), Tregs cultured with LV-ARsh-transfected Hepa1–6. Co-culture (isotype), Tregs cultured with Hepa1–6 cells in the presence of polyclonal goat IgG; Co-culture (Ab), Tregs cultured with Hepa1–6 cells in the presence of AR neutralizing antibody. *N* = 6 per group. Data presented as mean ± SD. *p < 0.05; **p < 0.01; ***p < 0.001.

Since HCC cells over express AR, we hypothesized that AR produced by HCC might be responsible for intratumoral Tregs activation. To test this hypothesis, we applied a non-contact co-culture system to culture intratumoral Tregs with Hepa1–6 cells, and evaluated the Treg signature gene expression by qRT-PCR. Hepa1–6 cells and Tregs were separated by the 0.4 μm pore polycarbonate membrane inserts to avoid direct cell contact. We found that the mRNA levels of CTLA-4 and ICOS in Tregs increased after co-culture with Hepa1–6 cells, as compared with Tregs cultured alone (Figure 3A). However, the expression of other gene including IL-10 and TGF-β was not significantly changed (Figure 3A), suggesting IL-10 and TGF-β expression might not be altered by Hepa1–6-derived soluble factors. To evaluate the role of AR in Hepa1–6-mediated Tregs activation, Hepa1–6 cells were transfected with lentivirus that carried AR shRNA (LV-ARsh) or scramble shRNA (LV-scramble) before co-culture with Tregs. In comparison with non-transfected cells, Hepa1–6 cells transfected with LV-ARsh showed low AR expression, while LV-scramble transfected Hepa1–6 cells and non-transfected cells expressed similar amount of AR protein (Figure 3B). Expression of other EGF family members such as EGF, TGF-α and epiregulin were not influenced by transfection of lentivirus (Figure 3B), suggesting the gene silencing was AR-specific. Co-culture of Tregs with lentivirus-transfected Hepa1–6 cells revealed that AR gene knockdown abolished Hepa1–6 mediated up-regulation of CTLA-4 and ICOS expression in Tregs (Figure 3C, 3D). To further confirm the effect of AR, we co-cultured intratumoral Tregs with Hepa1–6 cells as above but using an AR neutralizing antibody to block the function of AR. Consistently, the neutralizing antibody significantly restrained the effect of AR, demonstrated by lower expression of CTLA-4 and ICOS in comparison with simply co-cultured Tregs or the isotype antibody group (Figure 3E, 3F). Therefore, these results suggested that AR was involved in HCC mediated phenotypic change of Tregs.

### AR promotes Tregs activity to suppress anti-tumor immunity *in vitro*

**Figure 4:**
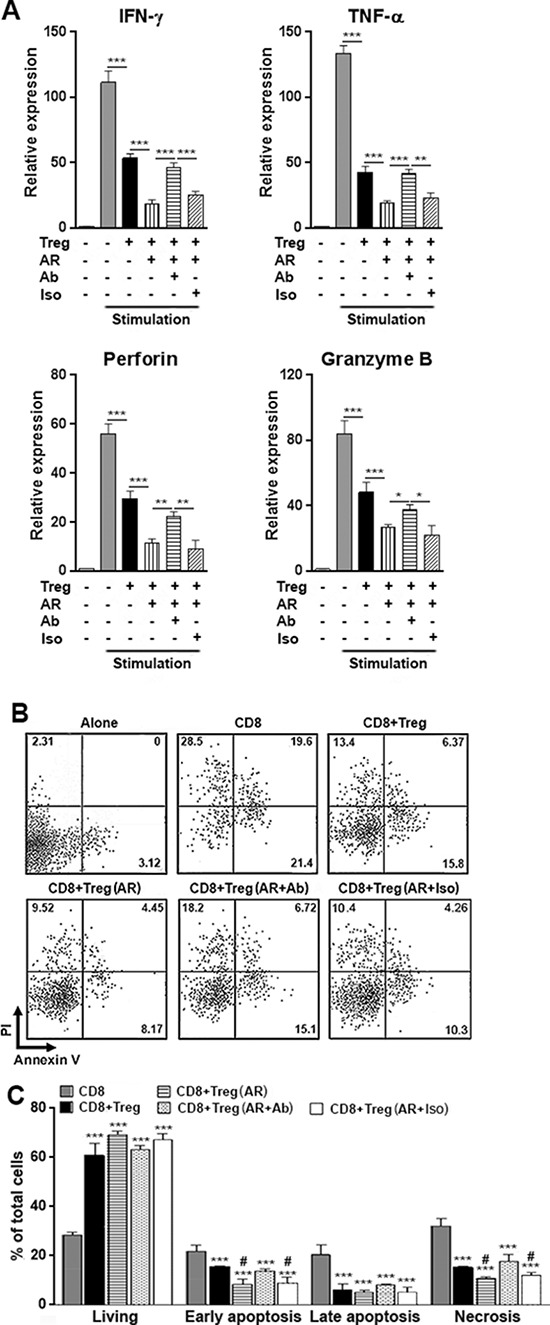
AR promotes Treg activity to suppress anti-tumor immunity *in vitro* **A.** Intratumoral CD8^+^ T cells and Tregs were isolated from Hepa1–6 xenografts and were co-cultured in the medium containing agonistic antibodies (anti-CD3 and anti-CD28). 100 ng/ml AR, 10 μg/ml AR neutralizing antibody or 10 μg/ml polyclonal goat IgG were present or absent in the co-culture. At day 4 after antibodies stimulation, CD8^+^ T cells were sorted by flow cytometry, and expression of IFN-γ, TNF-α, perforin and granzyme B were analyzed using qRT-PCR. *p < 0.05; **p < 0.01; ***p < 0.001. **B–C.** Intratumoral CD8^+^ T cells and Tregs were treated the same way as in (A) At day 4 after stimulation, CD8^+^ T cells were sorted by flow cytometry and were added into Hepa1–6 cells for additional 24 h incubation. Then CD8^−^ Hepa1–6 cells were stained with PI and Annexin V to analyze cell death. Representative dot plots of cell death is shown in (B) Numbers in the quadrants are the percentages of each cell population. Statistical analysis for cell death is shown in (C) Stimulation, stimulation with agonistic antibodies; Ab, AR neutralizing antibody; Iso, polyclonal goat IgG; Alone, Hepa1–6 cells cultured alone; CD8, Hepa1–6 cells cultured with CD8^+^ T cells; CD8+Treg, Hepa1–6 cells cultured with CD8^+^ T cells which were previously cultured with Tregs; CD8+Treg (AR), Hepa1–6 cells cultured with CD8^+^ T cells which were previously cultured with Tregs and AR; CD8+Treg (AR+Ab), Hepa1–6 cells cultured with CD8^+^ T cells which were previously cultured with Tregs and AR and AR neutralizing antibody. CD8+Treg (AR+Iso), Hepa1–6 cells cultured with CD8+ T cells which were previously cultured with Tregs and AR and polyclonal goat IgG. *N* = 6 per group. Data presented as mean ± SD. *p < 0.05; **p < 0.01; ***p < 0.001 compared with CD8 group. #*p* < 0.05 compared with CD8+Treg group.

Tregs inhibit anti-tumor immunity of CD8^+^ T cells [6–9]. To test whether AR mediated activation of Tregs is involved in regulating anti-tumor immunity, CD8^+^ T cells and Tregs were isolated from the Hepa1–6 xenografts and pooled. Tregs were co-cultured with CD8^+^ T cells with plate-bound anti-CD3 and soluble anti-CD28 antibody, in the presence or absence of AR and AR neutralizing antibody or polyclonal goat IgG. 4 days after anti-CD3 and anti-CD28 activation, CD8^+^ T cells were subject to qRT-PCR to analyze the expression of molecules that related to tumor-killing. As shown in Figure 4A, untreated Tregs effectively inhibited expression of IFN-γ, TNF-α, perforin and granzyme B. In the presence of AR, Tregs further decreased expression of above molecules, whereas the existence of AR neutralizing antibody diminished the effect of AR. It was noted that the expression of perforin and granzyme B were partially recovered by AR neutralizing antibody, suggesting other factors might also contribute to regulate Tregs activity. Since CD8^+^ T cells did not express EGFR, it was unlikely that AR directly inhibited CD8^+^ T cells. As the isotype control, polyclonal goat IgG did not significantly alter the result, suggesting the efficacy of the neutralizing antibody was AR-specific (Figure 4A). To test the possibility that other EGF family members such as EGF would induced similar changes to Tregs, we performed similar co-culture experiments using recombinant mouse EGF and neutralizing anti-EGF antibody as treatment. To our surprise, as shown in Supplemental Figure 1, EGF only mildly enhanced Treg activity to inhibit IFN-γ and TNF-α expression in CD8+ T cells, while it had no significant effect on perforin and granzyme B expression. Thus, AR and EGF might trigger non-uniform signaling in Tregs to modulate Treg function.

To evaluate the anti-tumor effect of CD8^+^ T cells, CD8^+^ T cells were sorted and added to Hepa1–6 cells for co-culture. Apoptosis of Hepa1–6 cells were determined 24 h after addition of CD8^+^ T cells. As shown in Figure 4B and 4C, CD8^+^ T cells potently induced hepa1–6 apoptosis and necrosis, whereas untreated Tregs strongly inhibited hepa1–6 cell death induced by CD8^+^ T cells. AR enhanced Tregs activity to further inhibit hepa1–6 cell death, and AR neutralizing antibody abolished the effect of AR. Therefore, AR mediated activation of Tregs contributed to the suppression of anti-tumor immunity.

### AR suppresses the anti-tumor activity of CD8^+^ T cells *in vivo*

**Figure 5:**
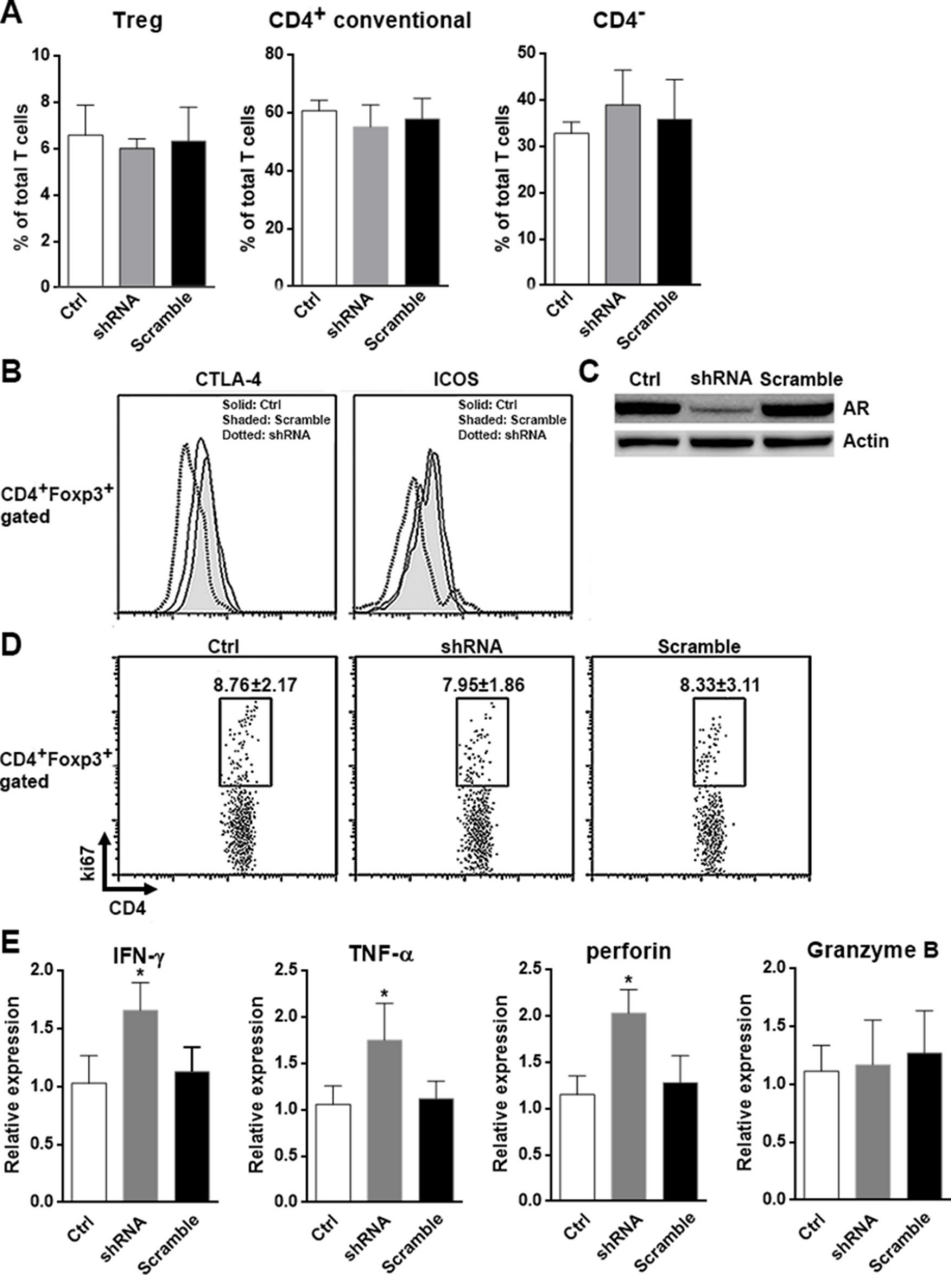
AR suppresses anti-tumor activity of CD8^+^ T cells *in vivo* **A.** Proportion of each T subset in the Hepa1–6 xenografts was determined by flow cytometry. **B.** Histograms of the expression of CTLA-4 and ICOS on intratumoral Tregs determined by flow cytometry. This is a representative of three independent experiments. **C.** AR expression in the xenografts was determined by Western blotting. This is a representative of two independent experiments. **D.** Intratumoral Tregs proliferation was determined by ki67 staining. Numbers in the plots were the percentages of ki67^+^ cells presented as mean ± SD. **E.** Expression of IFN-γ, TNF-α, perforin and granzyme B in intratumoral CD8^+^ T cells were analyzed using qRT-PCR. Ctrl, xenografts of non-transfected Hepa1–6 cells; shRNA, xenografts of LV-shRNA-transfected Hepa1–6 cells; Scramble, xenografts of LV-scramble-transfected Hepa1–6 cells. *N* = 7 per group. *p < 0.05.

To test whether the above observations could be repeated *in vivo*, we established mouse xenograft tumor models using Hepa1–6 cells transfected with LV-ARsh or LV-scramble. These cells were inoculated into Rag1^−/−^ mice. Four weeks after inoculation, intratumoral T cells in the xenografts were isolated and analyzed. Consistent with the role of AR in tumor growth, the size of LV-ARs xenografts were smaller (about 70%) than that of the size of LV-scramble xenografts or non-transfected xenografts (data not shown). However, the proportions of Tregs, CD4^+^ conventional T cells and CD4^−^ T cells were comparable among each group, suggesting AR knockdown did not alter the recruitment of each T cell subset into the tumor (Figure 5A). The expression of CTLA-4 and ICOS was decreased on Tregs in LV-ARsh xenografts, in comparison with their counterparts in non-transfected or LV-scramble xenografts (Figure 5B). The expression of CTLA-4 and ICOS on Tregs in non-transfected xenografts and LV-scramble transfected xenografts was comparable, suggesting transfection of lentivirus did not impact the phenotype of Treg (Figure 5B). AR knockdown in LV-ARsh xenografts was confirmed by Western blotting (Figure 5C). The Tregs proliferation in xenografts was quantitated by ki67 staining and no significant difference was seen among three groups, suggesting AR did not involve in modulating intratumoral Tregs proliferation (Figure 5D). In LV-ARsh xenografts, CD8^+^ T cells have higher expression of IFN-γ, TNF-α and perforin than those in non-transfected xenografts and LV-scramble xenografts, suggesting AR knockdown promoted anti-tumor activity of CD8^+^ T cells (Figure 5E). However, the expression of granzyme B was not changed, suggesting some factors might compensate for AR in the regulation of granzyme B *in vivo* (Figure 5E).

### AR promotes Treg activity through mTORC1 signaling

**Figure 6:**
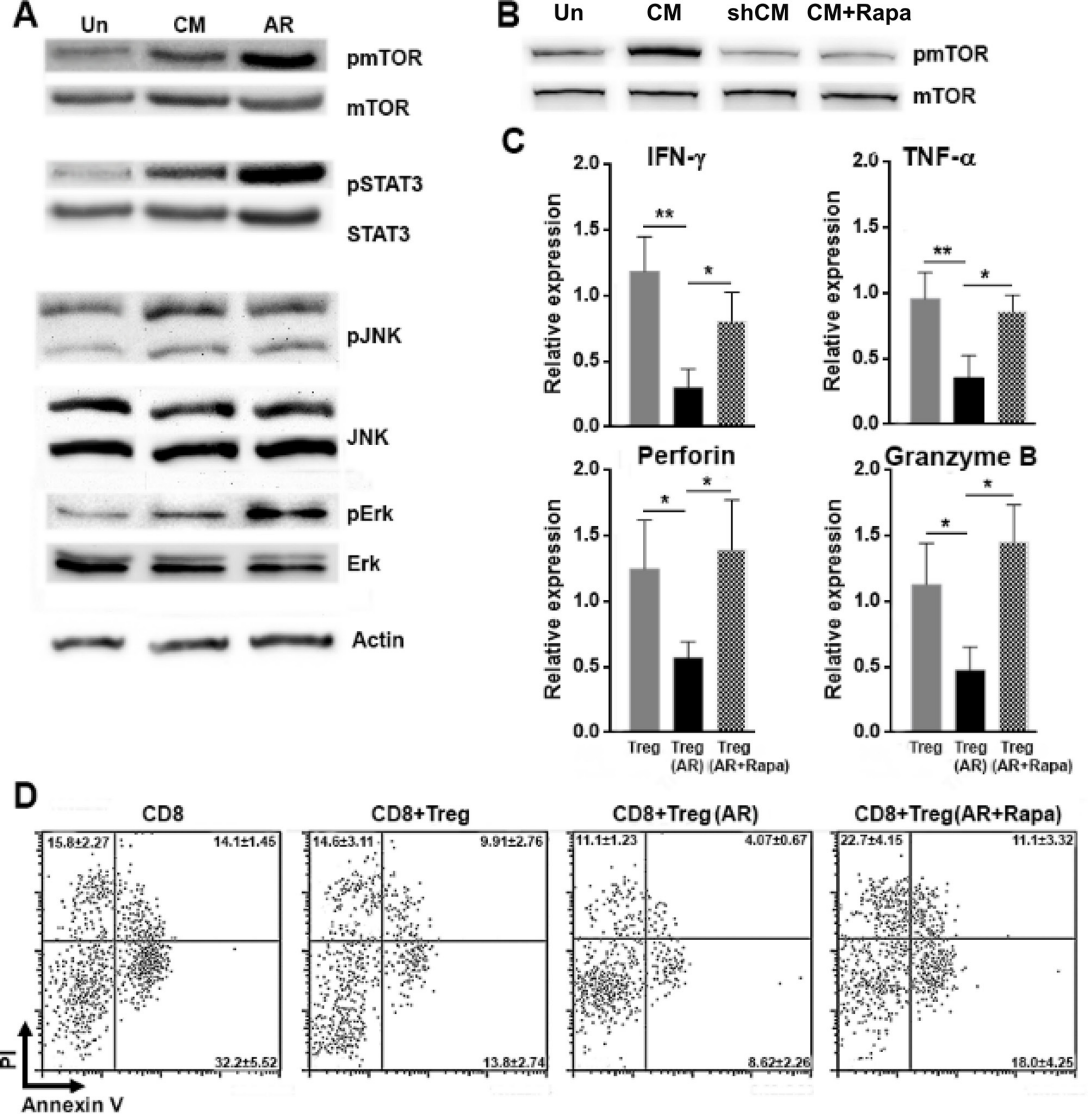
AR promotes Treg activity through mTORC1 signaling **A.** Tregs were cultured in Hepa1–6-conditioned medium or in the medium containing 100 ng/ml AR for 1 h. Phosphorylation of indicated signaling molecules determined by Western blotting. Un, untreated Tregs; CM, hepa1–6-conditioned medium; AR, AR-containing medium. **B.** mTOR phosphorylation in cultured Tregs determined by Western blotting. Un, untreated Tregs; CM, conditioned medium of non-transfected hepa1–6 cells; shCM, conditioned medium of LV-ARsh-transfected hepa1–6 cells; CM+Rapa, conditioned medium of non-transfected hepa1–6 cells with rapamycin. **C.** Tregs were treated with 100 ng/ml AR and 100 ng/ml rapamycin for 24 h. Tregs were then mixed with intratumoral CD8^+^ T cells at 1:1 and cultured for additional 24 h. Then the expression of IFN-γ, TNF-α, perforin and granzyme B in CD8^+^ T cells were analyzed using qRT-PCR. Treg, CD8^+^ T cells cultured with untreated Tregs; Treg (AR), CD8^+^ T cells cultured with Tregs pre-treated with AR; Treg (AR+Rapa), CD8^+^ T cells cultured with Tregs pre-treated with AR and rapamycin. **D.** The experimental procedure was the same as (C), except that after co-culture with Tregs, CD8^+^ T cells were sorted by flow cytometry and were added into Hepa1–6 cells. 24 h after addition of CD8^+^ T cells, Hepa1–6 cell death was determined. CD8, Hepa1–6 cells cultured with CD8^+^ T cells; CD8+Treg, Hepa1–6 cells cultured with CD8^+^ T cells which were previously cultured with Tregs; CD8+Treg (AR), Hepa1–6 cells cultured with CD8^+^ T cells which were previously cultured with AR-treated Tregs; CD8+Treg (AR+Rapa), Hepa1–6 cells cultured with CD8^+^ T cells which were previously cultured with AR-and-rapamycin-treated Tregs. *N* = 8 per group. Data presented as mean ± SD. *p < 0.05; **p < 0.01.

Previous studies showed that AR activates EGFR and mTORC1 signaling in different cell types [24–26]. More importantly, mTORC1 enhances Tregs function by controlling ICOS and CTLA-4 expression [27, 28]. However, to our knowledge, whether AR activates mTORC1 in Tregs has not been reported. Thus, we investigated mTORC1 and other signaling pathways downstream of AR using intratumoral Tregs treated with AR or hepa1–6-conditioned medium. We found that the level of phosphorylated mTOR, STAT3, JNK and Erk1/2 were elevated in Tregs treated with AR or hepa1–6-conditioned medium, compared with that in untreated Tregs (Figure 6A and Supplemental Figure 2). Furthermore, rapamycin, which preferentially blocks mTORC1 pathway, abolished mTOR phosphorylation induced by conditioned medium. Meanwhile, conditioned medium from LV-ARsh hepa1–6 cell culture failed to induce mTOR phosphorylation (Figure 6B). Thus, mTORC1 signaling was activated by AR in hepa1– 6-conditioned medium. To determine the role of AR-induced mTORC1 activation in regulating anti-tumor immunity, Tregs were treated with rapamycin and/or AR before co-culture with intratumoral CD8^+^ T cells. As shown in Figure 6C, rapamycin diminished the effect of AR, demonstrated by high expression of IFN-γ, TNF-α, perforin and granzyme B in comparison with AR-treated Tregs. Consistently, compared with AR-treated Tregs, rapamycin-treated Tregs were less capable of suppressing CD8^+^ T cells, and resulted in increased tumor cell death (Figure 6D).

### Blocking EGFR signaling in Tregs enhances anti-tumor immunity

**Figure 7:**
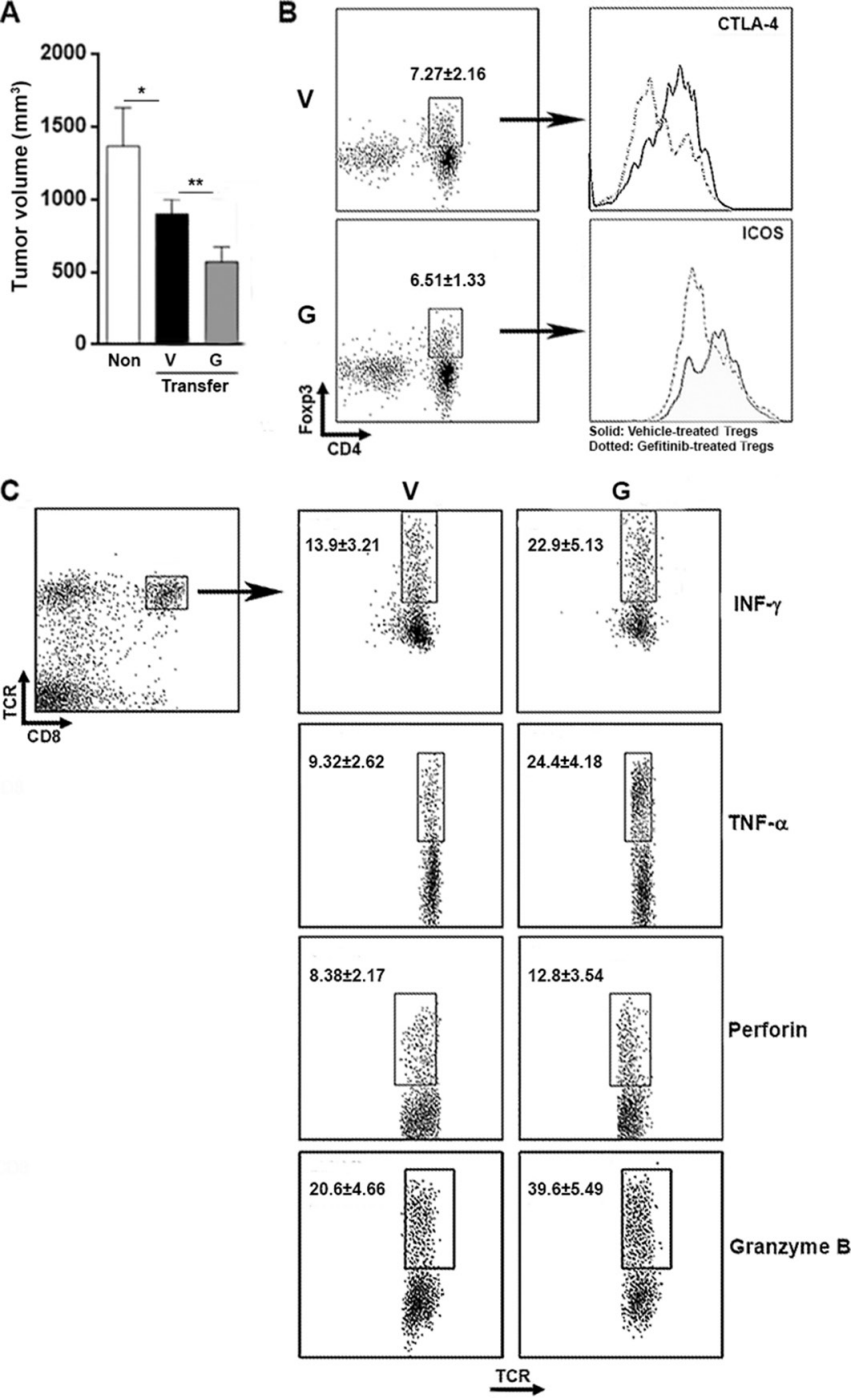
Inhibition of EGFR in Tregs leads to activation of T cells and tumor suppression **A.** Tumor xenograft size in recipient mice. Non, non-transferred mice; Transfer, mice transferred with T cells; V, mice transferred with vehicle-treated T cells; G, mice transferred with Gefitinib-treated T cells. **B.** Expression CTLA-4 and ICOS on Tregs. This is a representative data of three mice. V, mice transferred with vehicle-treated T cells; G, mice transferred with Gefitinib-treated T cells. **C.** Expression of IFN-γ, TNF-α, perforin and granzyme B in intrtumoral CD8^+^ T cells were determined by flow cytometry. Numbers in the plots are the percentages of gated positive cells presented as mean ± SD. *N* = 8 per group. V, mice transferred with vehicle-treated T cells; G, mice transferred with Gefitinib-treated T cells. *p < 0.05; **p < 0.01.

To investigate the relationship between AR and EGFR signaling in Tregs, we used Gefitinib, a selective EGFR tyrosine kinase inhibitor, to block the EGFR signaling. Gefitinib (100 ng/ml) was used to treat T cells for 24 h before T cells were transferred into Hepa1– 6-inoculated Rag1^−/−^ mice. As shown in Figure 7A, analysis of the xenograft tumor size revealed that mice receiving vehicle-treated T cells had significantly smaller tumors than non-transferred mice. Transfer of Gefitinib-treated T cells further reduced tumor size, suggesting that Gefitinib enhanced anti-tumor immunity. The proportion of intratumoral Tregs in total intratumoral T cells was not changed in mice receiving Gefitinib-treated T cells, suggesting Treg recruitment was not altered (Figure 7B). CTLA-4 and ICOS expression were low on intratumoral Tregs of mice receiving Gefitinib-treated T cells (Figure 7B). Meanwhile, compared with mice receiving vehicle-treated T cells, CD8^+^ T cells expressed high IFN-γ, TNF-α, perforin and granzyme B in mice receiving Gefitinib-treated T cells. (Figure 7C). Taken together, these data suggested that Gefitinib promoted anti-tumor immunity.

## DISCUSSION

HCC is the fifth most common cancer with continuously high mortality. Therefore, developing new therapeutic strategy is crucial to decrease recurrence rate and to improve the overall survival of HCC patients [29]. The rationale for immunotherapy is based on the findings that specific CD8^+^ T cell inhibits various tumor-associated antigens (TAAs) in HCC patients and T-cell infiltration in the tumor tissue has clinical benefit. Effector CD8^+^ T cells inhibit tumor growth through production of cytotoxic mediators. However, TAA-specific CD8^+^ T-cell response to HCC seems to be limited. It is believed that different mechanisms contribute to the failure of the cellular immune response [29]. CD4^+^CD25^+^Foxp3^+^ Tregs were considered as a crucial factor that suppress the anti-tumor function of Effector CD8^+^ T cells [7, 9, 30, 31]. However, how Treg activity is modulated in the tumor sites remains largely unexplored.

In this study we found that HCC cells suppress CD8^+^ T cell-mediated anti-tumor immunity through enhancing Treg activity. Our important finding is the role of AR in HCC cells-mediated immunomodulation. AR is synthesized as a transmembrane protein, and its extracellular domain is proteolytically processed to release the mature protein [32]. As an EGF family member, AR was shown to regulate growth of distinct cell types [12]. AR-producing cells include mast cells [33], basophils [34], dendritic cells [35], innate lymphoid cells [36], keratinocytes [37] and activated TH2 cells [38]. More importantly, AR is increasingly recognized as a potent oncogenic factor that overexpressed in different types of human cancers, specifically, downregulation of AR expression or blocking its function, have shown promising pre-clinical results, suggesting that these strategies could enhance the effectiveness of conventional antitumoral approaches [16, 39–42]. In HCC cells, AR has been intensely studied, overexpression of AR enhanced the proliferation rate, anchorage-independent growth, drug resistance, and *in vivo* tumorigenic potential of HCC cells, which emphasize the importance of AR in HCC development and treatment [43–47]. However, little is known about what factors drive the expression of AR in HCC cells. Our current study focused on dissecting the signaling pathway(s) related to AR expression.

Since CD8^+^ T cells are critical for inhibiting tumor growth, we first studied the role of HCC-derived AR in regulation of CD8^+^ T cells. Our result indicated that intratumoral CD8^+^ T cells do not express EGFR similar to their splenic counterparts [11]. Therefore, it is unlikely that AR directly regulates CD8^+^ T cells. A recent study indicates that AR plays an important role in regulating Treg cell function [10]. In liver diseases, Tregs protect livers from overwhelming damage, but also contribute to the compromise and even failure of CD8^+^ T cell response to infection and carcinoma [30, 48, 49]. We speculated that HCC cells express AR to modulate Treg cell activity and subsequently restrain anti-tumor immunity. As expected, we found that intratumoral Tregs up-regulated EGFR expression on their cell surface. More importantly, we found that HCC-derived AR enhanced Treg activity and in turn suppressed anti-tumor activity of intratumoral CD8^+^ T cells. Addition of AR neutralizing antibody, or down-regulating AR expression in HCC cells, diminished the enhancement of Tregs activity by HCC cells. To our knowledge, we are the first to report the role of AR in modulating immunity in HCC. AR plays a crucial role in establishing interactions among HCC cells, Tregs and effector CD8^+^ T cells. However, what caused the up-regulation of EGFR expression in intratumoral Tregs is still a puzzle. EGFR receptors include AR, EGF, TGF-α, epiregulin, heparin-binding EGF (HB-EGF) and Hepatocarcinoma cell-derived hepatoma-derived growth factor (HDGF), have been confirmed to mediate tumorigenesis and promote tumor progression [50, 51]. Since splenic Tregs have low EGFR, it is likely that intratumoral milieu induced high EGFR expression in infiltrating Tregs. Whether the high EGFR expression is caused by these soluble factors or direct cell contact is an unanswered question and needs further study.

As an EGF family member, AR is supposed to bind to EGFR and induce intracellular signaling in target cells. One of the EGFR downstream signaling cascade is the mTORC1 signaling pathway, which regulates cell growth, differentiation, senescence and metabolism [52]. The role of mTOR in regulating immune response has been a research interest for the past decade. Recent research indicated that mTORC1 regulates Treg cell homeostasis and function [27, 28, 53]. We therefore speculated that AR triggers mTORC1 signaling in Tregs to modulate Tregs function. Our results confirmed this speculation. Both AR and HCC cell-conditioned medium induced activation of phosphorylated mTOR. Furthermore, inhibition of mTOR activation by rapamycin blocked AR-induced enhancement of Treg cell activity. We suggested that AR promotes Treg cell function through activation of mTORC1 signaling in Tregs. It was noted that other molecules in EGFR signaling pathways such as STAT3, JNK and Erk were also activated in AR-treated Tregs. Whether these molecules are involved in modulating intratumoral Treg activity remains unclear. It could be one of our future plans to dissect how these molecules influence Tregs.

Taken together, our *in vitro* and *in vivo* studies demonstrated that HCC cells over express AR and promote Treg cell activity, leading to suppress CD8^+^ T cell-mediated anti-tumor response. Although it is not clear whether the interactions among HCC cells, Tregs and CD8^+^ T cells present in human HCC patients, our study sheds some light in immunomodulation and provides a rational for designing immunotherapy for HCC.

## MATERIALS AND METHODS

### Mouse HCC xenograft model and adoptive transfer of T cells

All animal experiments were conducted in compliance with institutional guidelines and Wuhan University Guidelines for the Use of Animals. All animal procedures were approved by Wuhan University School of Medicine Animal Care and Use Committee. Six-to-eight week old wild type C57BL/6J and Rag1^−/−^ male mice were purchased from Nanjing Biomedical Research Institute of Nanjing University (Nanjing, China). Rag1^−/−^ mice were subcutaneously (s.c.) inoculated with 1 × 10^6^ Hepa1–6 cells at the left flank. At 28 days after inoculation, the mice were sacrificed by inhalation of carbon dioxide for an average of 5 min. Tumor volume was measured according to the standard formula 1/2 × L × W^2^. In some experiments, 2 × 10^7^ splenic CD3^+^ T cells were sorted from C57BL/6J mice by flow cytometry and were i.v. injected into Hepa1–6-inoculated mice twice a week from day 7 to day 28 after inoculation.

### Isolating intratumoral T cells

Tumor-infiltrating immune cells were isolated as previously described with some modifications [18, 19]. Briefly, fresh tumor tissues were cut into small pieces and digested at 37°C for at least 20 min in RPMI 1640 supplemented with 0.05% collagenase Type IV (Sigma-Aldrich, St. Louis, MO), 0.002% DNase I (Roche, Basel, Switzerland) and 20% fetal calf serum (FCS, HyClone Laboratories, Logan, UT). Dissociated cells were then filtered through a 150-μm mesh and mononuclear cells were obtained by Ficoll density gradient centrifugation. Mononuclear cells were collected from the interface, washed in PBS, and resuspended in Tris-NH_4_Cl solution to lyse residual red blood cells. Cells were washed with PBS twice and were subjected to further processing. Intratumoral CD4^+^CD25^+^ T cells (Treg-enriched cells) were enriched from intratumoral mononuclear cells using the EasySep™ Mouse CD25 Regulatory T Cell Positive Selection Kit (Stemcell Technologies) following the manufacturer's instructions. Foxp3 staining was conducted to confirm that more than 80% enriched cells were Foxp3^+^ cells. Intratumoral CD8^+^ T cells were selected using EasySep™ Mouse CD8^+^ T Cell Enrichment Kit (Stemcell Technology). Before further experiments, Treg-enriched cells and CD8^+^ T cells were cultured in RPMI 1640 containing 10% FCS, 2 mM L-glutamine, 100 U/ml penicillin and 100 μg/ml streptomycin.

### *In vitro* cell culture

Murine HCC cell lines Hepa1–6, Hepa-1c1c7, BpRC2 and c12 were cultured in 10-cm culture dishes in DMEM supplemented with 10% FCS, 1% penicillin-streptomycin, 1% HEPES, and 0.05 mM 2-ME (2-Mercapto-Ethanol) in an incubator at 37°C.

The culture of mouse primary hepatocytes was conducted following the established protocol with a few modifications [20]. Briefly, hepatocytes were isolated from livers of C57BL/6 mice by 0.1% collagenase IV (Sigma-Aldrich) digestion. The dissociated cells were then plated in collagen-coated 24-well or 6-well culture dishes with supplemented DMEM as described above. After culture for 4 h, the medium was replaced by fresh culture medium.

For Tregs and Hepa1–6 cells co-culture, Corning HTS Transwell 24-well plate was used (0.4 μm pore; Corning Costar). 5 × 10^5^ Hepa1–6 cells were seeded into the lower chamber and 1 × 10^5^ tumor-infiltrating Tregs were added in the upper chamber. After 24 h incubation, Tregs in the upper chamber were collected and subject to RNA extraction or flow cytometry. To detect the immunosuppressive effect of Tregs on CD8^+^ T cells, a 48-well plate was pre-coated with 5 μg/ml anti-CD3 monoclonal antibody (17A2, eBiscience). 1 × 10^6^ intratumoral CD8^+^ T cells and 2 × 10^5^ intratumoral Tregs were seeded into pre-coated wells in the presence of 2 μg/ml anti-CD28 antibody (37.51, eBioscience). Four days after stimulation, cells were stained with PE-Cy7 anti-CD8a antibody and CD8^+^ T cells were sorted by flow cytometry. Cytokine production in CD8^+^ T cells was measured by qRT-PCR. In some experiments, Tregs were treated with 100 ng/ml recombinant mouse AR protein (R&D systems), 10 μg/ml AR neutralizing antibody (R&D systems), 10 μg/ml polyclonal goat IgG (R&D systems) or 100 ng/ml rapamycin (EMD Millipore).

To determine the anti-tumor activity of CD8^+^ T cells, intratumoral CD8^+^ T cells were co-cultured with Hepa1–6 cells for 24 h. The ratio between CD8^+^ T cells and Hepa1–6 cells was 2:1. Cells were then dissociated with 10 mM EDTA-PBS at room temperature for 10 min before staining with 2 μg/ml Propidium iodide (PI), FITC Annexin V and PE-Cy7 anti-CD8a (53–6.7) (All from BD Biosciences) according to the manufacturer's instruction. Cell death of CD8a^−^ tumor cells was analyzed by flow cytometry within 1 h. Cell death can be divided into early apoptotsis (Annexin V^+^PI^−^), Late apoptotsis (Annexin V^+^PI^+^), and necrosis (Annexin V^−^PI^+^).

### Flow cytometry analysis

The following anti-mouse antibodies were used for detection and enrichment of immune cells: APC anti-CD3 (17A2), PE anti-TCRβ (H57–597), APC-Cy7 anti-CD4 (GK1.5), Alexa Fluor^®^ 488 anti-Foxp3 (R16–715), and PE-Cy7 anti-CD8a (53–6.7) (BD Pharmingen); PE anti-CTLA-4 (UC10–4B9) and PE anti-ICOS (7E.17G9) (eBioscience). PE anti-Ki67 (Biolegend), Anti-EGFR antibody (ab30) (Abcam). For cell surface staining, cells were incubated with corresponding antibodies in PBS for 15 min on ice before analysis on a FACSAria™ III cell sorter (FACSDiva software, BD). Dead cells that stained by propidium iodide (2 μg/ml) were excluded. For intracellular cytokine staining, cells were fixed and permeabilized with BD Cytofix/perm and Perm/wash buffer according to the manufacturer's protocol. Then cells were stained at room temperature for 30 min with Alexa Fluor^®^ 488 anti-IFN-γ (XMG1.2, Biolegend), Alexa Fluor^®^ 647 anti-TNF-α (MP6-XT22, Biolegend), APC anti-perforin (eBioOMAK-D, eBioscience) and PE anti-granzyme B (NGZB, eBioscience) respectively before analysis on a BD LSRII flow cytometer. For Foxp3 staining, Foxp3 fix/perm buffer (Biolegend) set was used according to the manufacturer's instruction. All flow cytometry data was analyzed with Flowjo 7.6.1 software. Cell sorting was performed on a FACSAria™ III cell sorter (FACSDiva software, BD) based on cell surface marker staining.

### RNA isolation, reverse transcription and quantitative RT-PCR (qRT-PCR)

Total RNA was isolated from cells using the RNeasy Mini Kit (Qiagen). One microgram of total RNA from each sample was transcribed into cDNA using SuperScript^®^ III First-Strand Synthesis System (Invitrogen) according to the manufacturers’ instruction. qRT-PCR was performed using Fast SYBR^®^ Green Master Mix (Invitrogen) on a 7300 qRT-PCR System (Invitrogen). Data was analyzed with 7300 system software. Primer sequences for each gene are as follows: β-actin (5′-AGAGGGAAATCGTGCGTGAC-3′ and 5′-CAATAGTGATGACCTGGCCGT-3′). AR (5′-ACTGTGCATGCCATTGCCTA-3′ and 5′-ACTGGGCATCTGGAACCATC-3′). IL-10 (5′-GATGCCTTCAGCAGAGTG AA-3′ and 5′-GCAACCCAGGTAACCCTTAAA-3′). TGF-β (5′-TGACGTCACTGG AGTTGTACGG-3′ and 5′-GGTTCATGTCATGGATGGTGC-3′). CTLA-4 (5′-ATGGC TTGTCTTGGACTCCG-3′ and 5′-ACCACTGAAGGTTGGGTCAC-3′). ICOS (5′-TGA CCCACCTCCTTTTCAAG-3′ and 5′-TTAGGGTCATGCACACTGGA-3′). LAG-3 (Lymphocyte-activation gene 3) (5′-GGCTGTGTCCTCACCTACAG-3′ and 5′-CCTAGAACCTTCAGCAGCGT-3′). CD73 (5′-TTCGAGGTGTGGACATCGTG-3′ and 5′-GTCCATCATCTGCGGTGACT-3′). Perforin (5′-CTGGCAGGGACGATGACCT-3′ and 5′-GGGAACCAGACTTGGGAGC-3′). Granzyme B (5′-ATCAAGGATCAGCAGC CTGA-3′ and 5′-TGATGTCATTGGAG AATGTCT-3′). IFN-γ (5′-TGAACGCTACA CACTGCATCTTGG-3′ and 5′-CGACTCCTTTTCCGCTTCCTGAG-3′). TNF-α (5′-GC CTCTTCTCATTCCTGCTTG-3′ and 5′-CTGATGAGAGGGAGGCCATT-3′). PCR conditions used for all primer sets were as follows: 95°C hot start for 10 min, followed by 40 amplification cycles of 95°C for 30 s (denaturing), 60°C for 1 min (annealing, extension and detection). Relative abundance of RNA was analyzed using 2^−ΔΔCt^ method.

### Enzyme linked immunosorbent assay (ELISA)

Fifty milligrams of normal mouse liver or Hepa1–6 xenograft tissues were cut into small pieces and homogenized manually in a homogenizer containing 0.25 ml of homogenization buffer (PBS containing 0.05% sodium azide, 0.5% Triton X-100, and protease inhibitor cocktail purchased from Roche, pH 7.2, 4°C) and then sonicated on ice for 10 minutes. Homogenates were centrifuged at 12,000 g for 10 minutes and supernatant was collected for ELISA. Cell culture supernatants were collected and stored at −80°C until used. AR concentration was determined by Mouse Amphiregulin DuoSet (R&D Systems) according to the manufacturer's protocol. The plates were read on a SpectraMax^®^ i3x microplate reader (Molecular Devices, Sunnyvale, CA).

### Western blotting

Western blotting was performed using the protocol as previously described [18]. The following antibodies were used: anti-β-actin, anti-AR (G-4) and anti-TGF-α (D-6) (Santa Cruz Biotechnology); anti-EGF (4E11) (Life Technologies); anti-phospho-mTOR (Ser2448), anti-mTOR, anti-phospho-STAT3 (Tyr705), anti-STAT3, anti-phospho-JNK (Thr183/Tyr185), anti-JNK, anti-phospho-Erk (Thr202/Tyr204) and anti-Erk (Cell Signaling Technology). Membranes were developed with SuperSignal West Pico Chemiluminescent Substrate (Thermo Scientific) and the optical density was analyzed using a UVP Bioimaging system (UVP, Upland, CA).

### Knockdown of AR expression by lentivirus transfection

Lentiviral particles containing mouse Amphiregulin shRNA or scramble shRNA were purchased from Santa Cruz Biotechnology. Transfection of Hepa1–6 cells with lentivirus was conducted following the manufacturer's instruction. Briefly, Hepa1–6 cells were plated into 24-well plate 24 h before transfection or until cells reached 50% confluent. On the day of transfection, the medium was replaced by fresh medium containing 5 μg/ml polybrene. Lentiviral particles were added into cell culture at the multiplicity of infection (MOI) of 10 and were incubated with cells overnight. Then the virus-containing medium was replaced by polybrene-free fresh medium and cells were incubated for another 36–48 h to allow for expression of puromycin-resistant gene. To select stable clones expressing shRNA, 2 μg/ml puromycin was added into cell culture and was refreshed every 3 days until resistant clones were identified. Resistant clones were expanded and AR expression was analyzed by Western blotting.

### Statistics

The data were analyzed by Prism 5.0 software (GraphPad). Quantitative data were expressed as mean ± SEM from the indicated number of experiments. Student's *t* test or one-way ANOVA were used for comparison of mean between the groups. *P* values < 0.05 were considered significant.

## SUPPLEMENTARY MATERIAL FIGURES


