# Supplementary material for: Amphiregulin activates regulatory T lymphocytes and suppresses CD8+ T cell-mediated anti-tumor response in hepatocellular carcinoma cells
Source: Oncotarget. 2015 Oct 6;6(31):32138–53. doi: 10.18632/oncotarget.5171 (PMC4741664; doi:10.18632/oncotarget.5171)
Supplement: Supplementary file 1 [file oncotarget-06-32138-s001.pdf]

SUPPLEMENTARY FIGURES

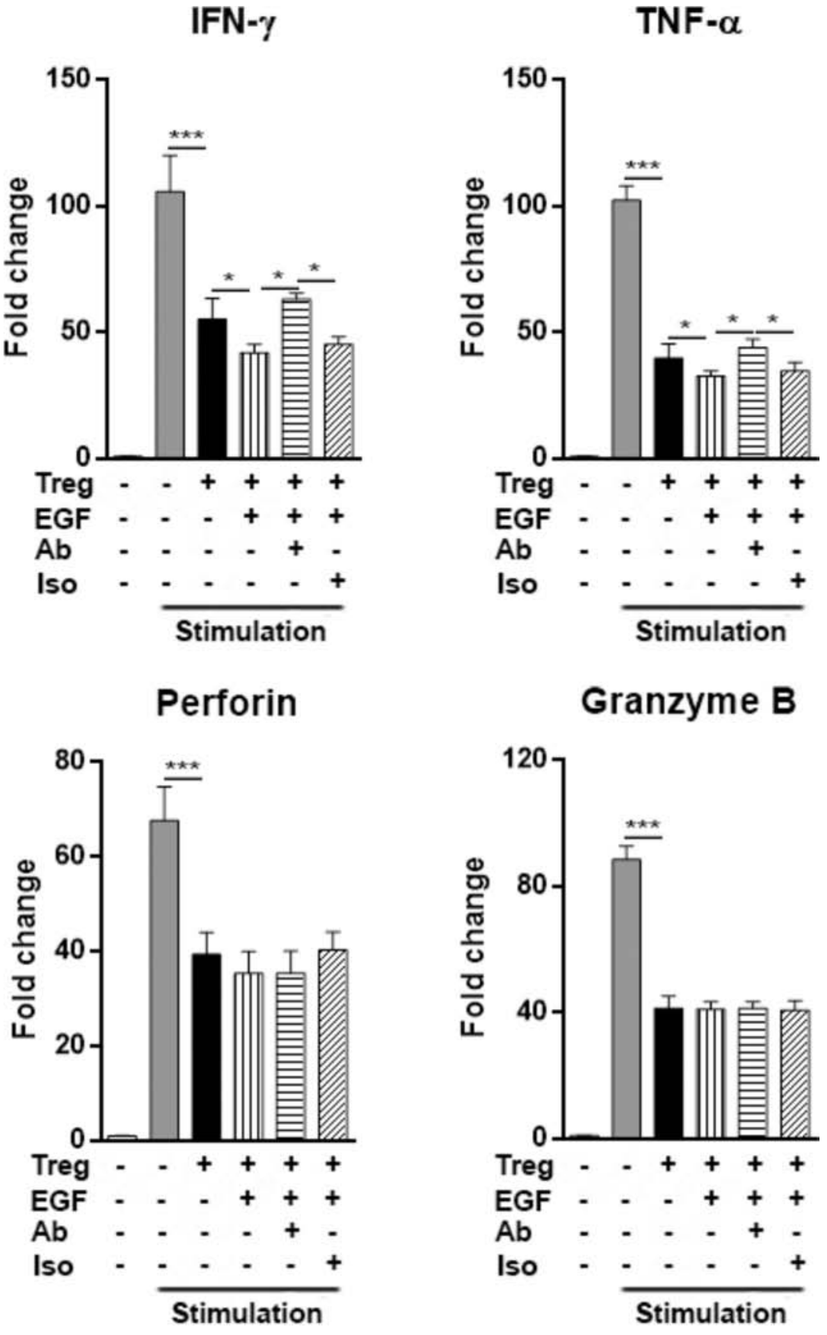

Supplementary Figure S1: EGF mildly modulate Treg activity on CD8+ T cells *in vitro*. Tumor-infiltrating CD8+ T cells and Tregs were isolated from Hepa1–6 xenografts and were co-cultured in the medium containing agonistic antibodies. 30 ng/ml mouse EGF, 10 µg/ml neutralizing anti-mouse EGF antibody or 10 µg/ml polyclonal goat IgG (All from R&D Systems) were present or absent in the co-culture. Note that the amount of EGF was equivalent to the molar concentration of 100 ng/ml AR, based on the molecular mass of their major soluble forms. At day 4 after stimulation, CD8+ T cells were sorted by flow cytometry, and expression of IFN-γ, TNF-α, perforin and granzyme B were analyzed using qRT-PCR. *$p < 0.05$; ***$p < 0.001$.

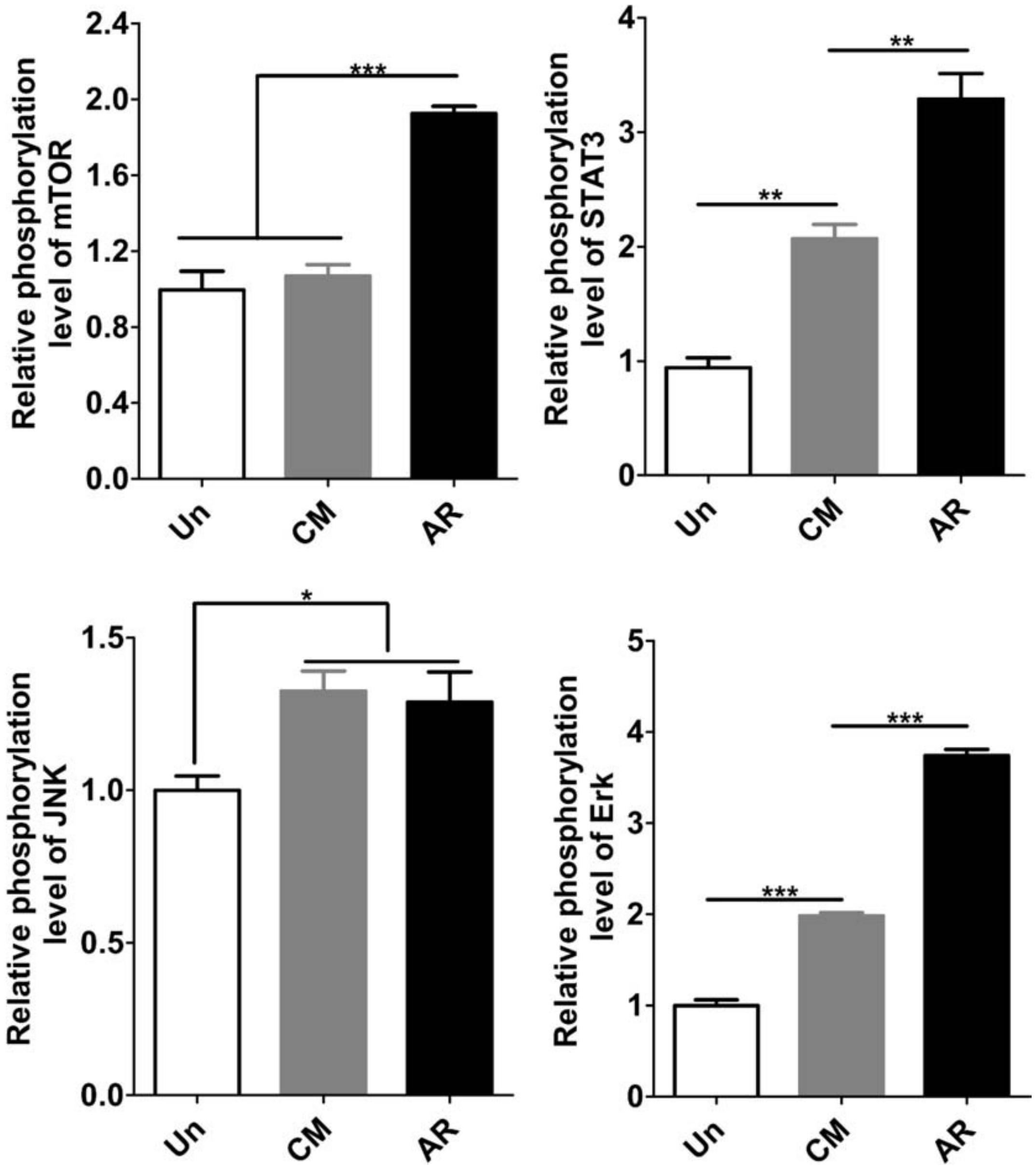

Supplementary Figure S2: Quantification analysis of mTOR, STAT3, JNK and Erk phosphorylation. As showed in Figure 6A, Tregs were cultured in Hepa1–6-conditioned medium or in the medium containing 100 ng/ml AR for 1 h. Phosphorylation of indicated signaling molecules determined by Western blotting. The grayscale of each band that exhibited in Figure 6A was analyzed. Un, untreated Tregs; CM, hepa1–6-conditioned medium; AR, AR-containing medium. $N = 3$ per group. Data presented as mean ± SD. $^{*}p < 0.05$; $^{**}p < 0.01$; $^{***}p < 0.001$.
